# Supplementary material for: Coordinated regulation of Mdr1- and Cdr1-mediated protection from antifungals by the Mrr1 transcription factor in emerging Candida spp
Source: mBio. 2025 Oct 7;16(11):e01323-25. doi: 10.1128/mbio.01323-25 (PMC12607694; doi:10.1128/mbio.01323-25)
Supplement: Supplemental Figures — Figures S1-S6. [file mbio.01323-25-s0006.pdf]

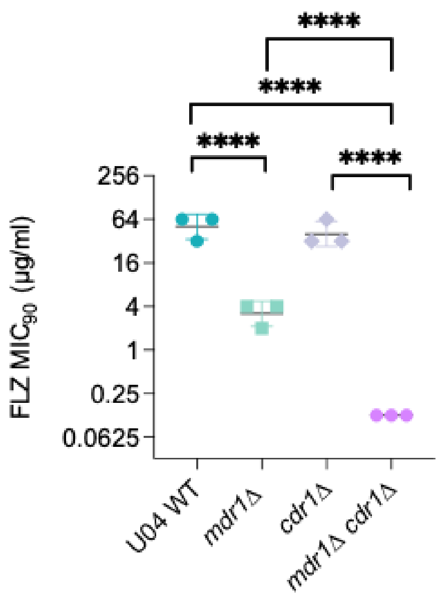

Figure S1: Effects of constitutive Mrr1 activity and *MDR1* and *CDR1* on susceptibility to fluconazole. Mean ± SD of FLZ MIC_{90} values of the U04 clinical isolate (native allele $MRR1^{Y813C}$) and its *mdr1Δcdr1Δ* mutant derivative, and the *mdr1Δ* and *cdr1Δ* mutants in the U04 *mrr1Δ*+$MRR1^{Y813C}$ background from three independent experiments performed on different days is shown. Ordinary one-way ANOVA and Tukey's multiple comparisons testing with a single pooled variance were used to evaluate the statistical significance of log2 transformed MIC values between strains. All significant comparisons are shown; ****, p<0.0001.

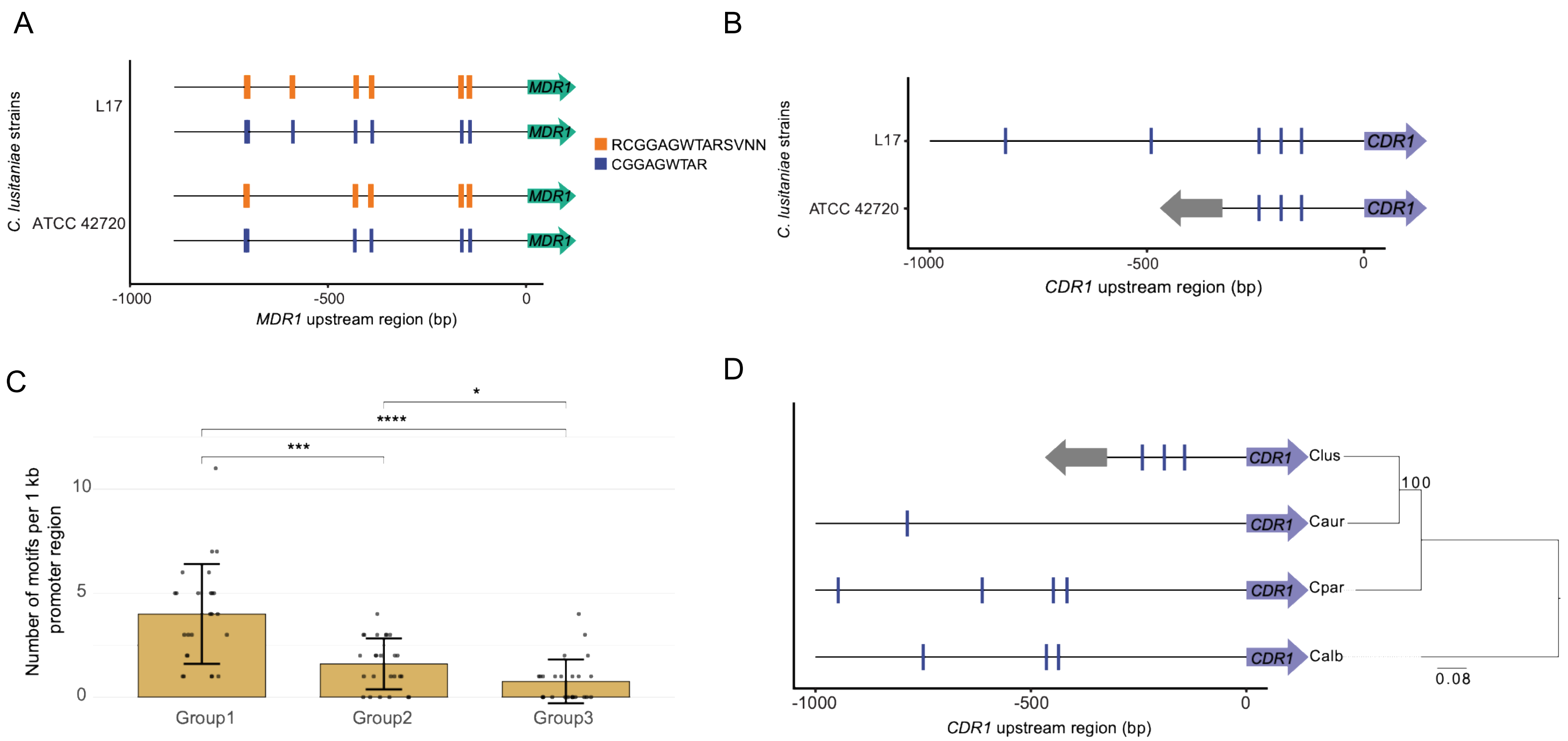

Figure S2: Consensus Mrr1-binding DNA motif in the promoter regions of Mrr1 targets. (A) The positions of the originally identified 14-nt Mrr1-binding motifs (orange hatches) and the 9-nt consensus Mrr1-binding motifs (cMBM; blue hatches) colocalize in the ~890 bp upstream intergenic regions of *MDR1* in *C. lusitaniae* L17 *and* ATCC 42720 strains. (B) cMBM location in the 1 kb upstream intergenic regions of *CDR1* from *C. lusitaniae* L17 *and* ATCC 42720. (C) Number of cMBMs found in the 1 kb upstream intergenic regions of genes in the following three groups: Group-1, the 25 genes that have a CUT&RUN peak and that were differentially expressed (DE) (Fig. 4A & Table S1A)*;* Group-2, genes with no CUT&RUN peak but were DE in RNA-seq analysis (File S2A); Group-3, genes not represented in both CUT&RUN and RNA-seq datasets. Each group had 25 genes (Table S5). Mean ± SD of each group shown. Pairwise comparisons were performed using Wilcoxon rank sum test; *,p<0.05, ***, p<0.001, ****, p<0.0001. (D) cMBM location in the 1 kb upstream intergenic regions of the *CDR1* homologs of *C. parapsilosis* CDC317, *C. auris* B8441*, C. albicans* SC5314 and *C. lusitaniae* ATCC 42720. The phylogenetic tree was constructed using the *CDR1* nucleotide sequences. (B, D) The intergenic region upstream of *CDR1* in ATCC 42720 is 326 bp. Grey arrow indicates the adjacent ORF *CLUG_03114.*

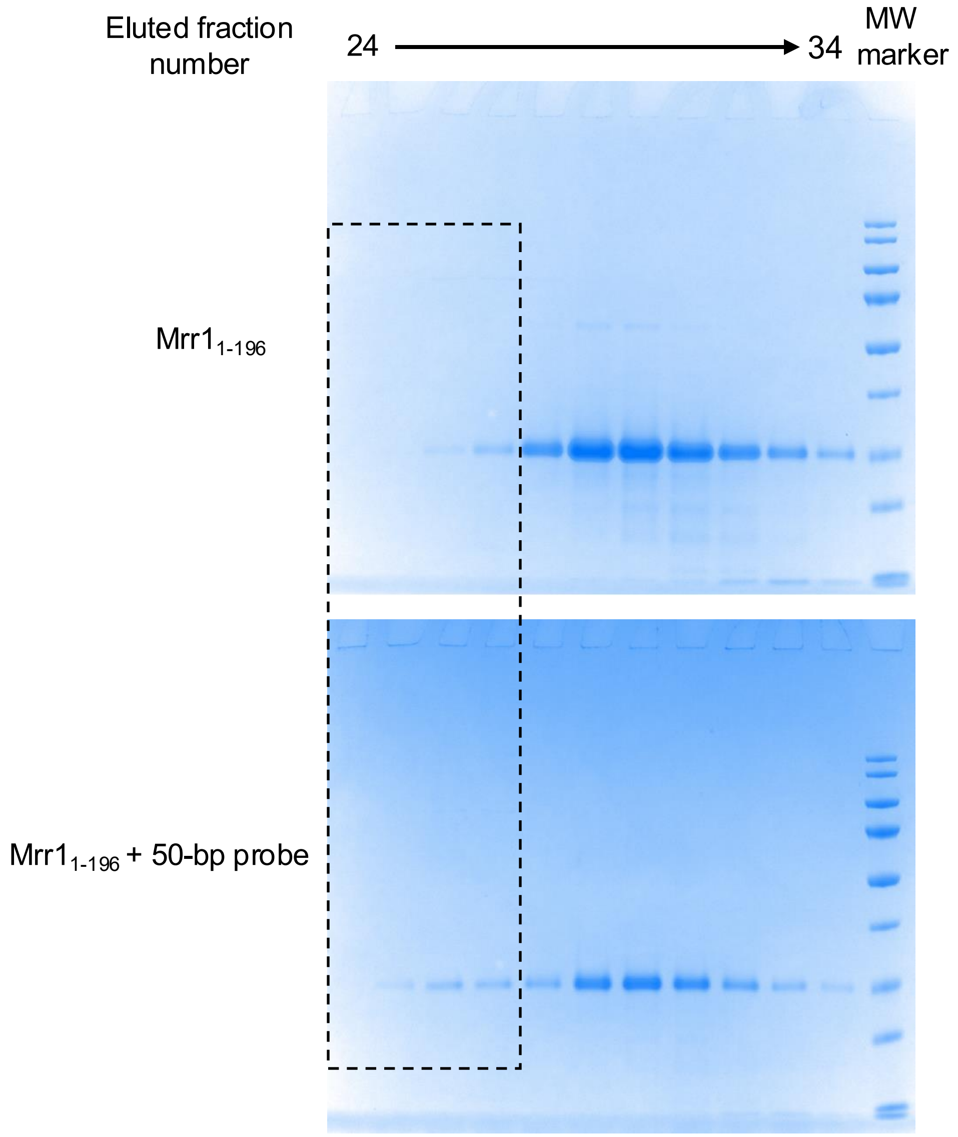

Figure S3: SDS-PAGE analysis of eluted fractions from size-exclusion chromatography (SEC). Eluted fractions from SEC corresponding to elution volumes between 14-20 ml in Fig. 5D. Fractions from Mrr1$_{1-196}$ only, and the Mrr1$_{1-196}$ and 50-bp probe mixture were analyzed. The boxed region corresponds to the elution volume where the peak corresponding to Mrr1$_{1-196}$-50-bp probe complex was detected in the chromatogram in Fig. 5D.

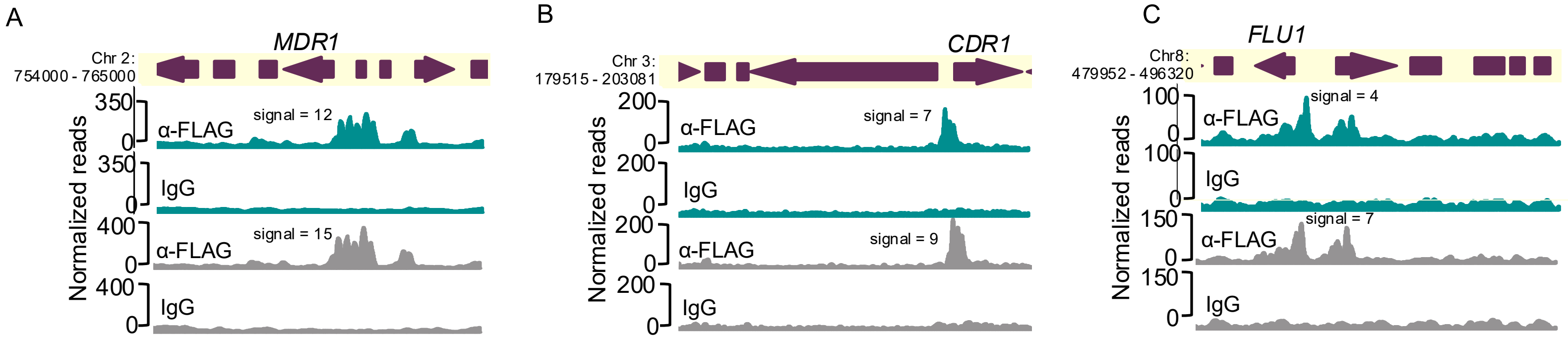

Figure S4: Comparison of binding profiles of constitutively active and low-activity Mrr1.
(A-C) Comparison of data from Figures 1D-F and Figures 5A-C to highlight the similarities in peak profiles. HF-Mrr1^{Y813C} (in blue) and HF-Mrr1^{ancestral} (in grey) CUT&RUN read coverage plots normalized per 20 bp bin size. Chromosomal positions of regions containing *MDR1, CDR1* and *FLU1* and adjacent genes are represented to scale with boxes and arrows. Peaks from HF-Mrr1-bound DNA recovered by an α-FLAG antibody and for the non-specific binding control recovered via IgG are shown. Signal indicates the average read density in α-FLAG relative to IgG within the peak region.

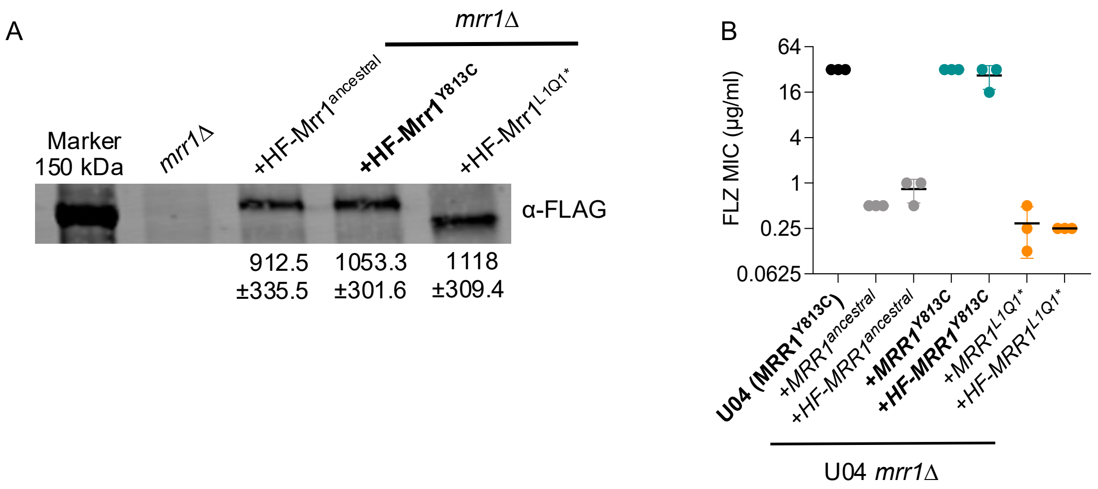

Figure S5: Biochemical and phenotypic analysis of HF-tagged Mrr1 variants. (A) Western blot of whole cell protein lysates of U04 strains expressing N-terminal 6xHis-3xFLAG-tagged Mrr1 (HF-Mrr1) variants. HF-Mrr1 was probed using an α-FLAG antibody. Mean ± SD of HF-Mrr1 band intensities normalized to total protein (n= 4 biological replicates). (B) FLZ MIC of U04 clinical isolate (native allele *MRR1*^{Y813C}) and U04 *mrr1*Δ complemented with untagged or *HF-MRR1* was determined by broth microdilution assays. The data shown represent the mean ± SD from three independent experiments. No more than a two-fold difference in MICs observed between data from strains with untagged Mrr1 variants and data from strains with their respective HF-tagged counterparts. Strains with constitutive Mrr1 activity are in bold.

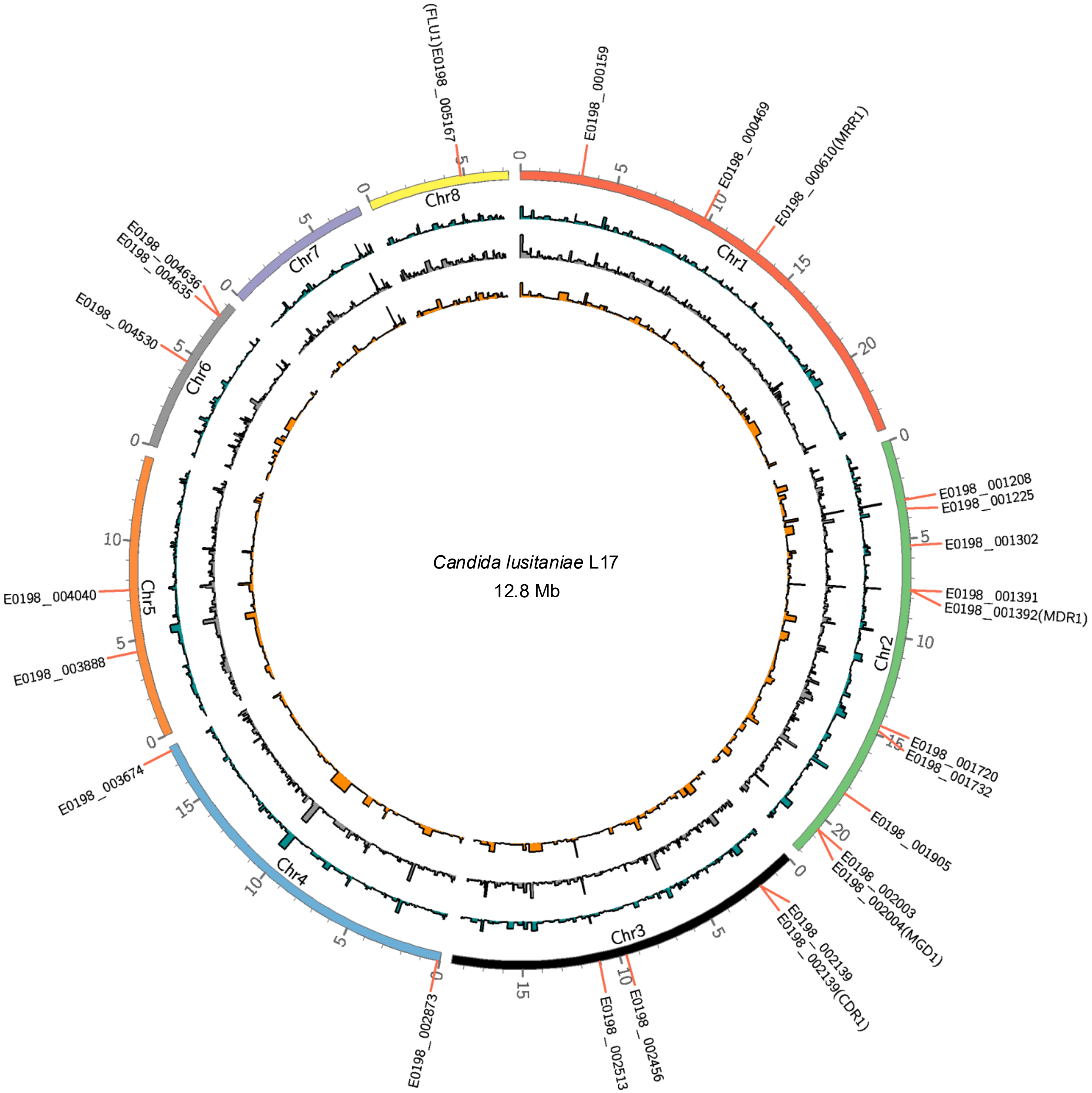

Figure S6: Global binding profiles of constitutively active and low-activity Mrr1. Circos plot showing global CUT&RUN-determined Mrr1-binding peaks of HF-Mrr1^{Y813C} (in blue), HF-Mrr1^{ancestral} (in grey) and HF-Mrr1^{L1Q1*} (in orange) in the *C. lusitaniae* L17 genome. Mrr1-binding peaks with a signal ≥2-fold compared to their respective IgG backgrounds and up to 1 kb away from the nearest ORF from Experiment 1 (see Supplemental Files 1, 4 and 5) were used. The genomic positions of the 25 differentially expressed genes that constitute the Mrr1-regulon are marked with the L17 gene IDs.
