# Supplementary material for: Coordinated regulation of Mdr1- and Cdr1-mediated protection from antifungals by the Mrr1 transcription factor in emerging Candida spp
Source: mBio. 2025 Oct 7;16(11):e01323-25. doi: 10.1128/mbio.01323-25 (PMC12607694; doi:10.1128/mbio.01323-25)
Supplement: Supplemental Tables — Tables S1-S5. [file mbio.01323-25-s0007.pdf]

Supplemental Table 1A. The Mrr1 regulon defined as genes that have HF-Mrr1^{Y813C} peaks in adjacent intergenic regions and show differential expression in RNA-seq comparisons of strains with constitutively active Mrr1 compared to strains with low Mrr1 activity or strains lacking *MRR1* in Demers *et al.* (1). Genes listed as upregulated and downregulated are in red and blue, respectively (1). The *C. lusitaniae* ATCC and L17 gene IDs along with their *C. albicans* homologs and their description are shown in the table. Log2 fold change (FC) of the differentially expressed genes are shown (1). *C. albicans* homologs and their description were obtained from Candida Genome Database (CGD). In the absence of a clear *C. albicans* homolog, homologs in *C. auris* are listed.

Column 5: Genes that were also differentially expressed in the comparison of two *C. lusitaniae* clinical isolates P1 and P3 with low and constitutive Mrr1 activity, respectively, in Kannan *et al.*(2). Upregulated indicates transcripts that were more abundant in P3.

***C. lusitaniae* ATCC Gene #**	***C. lusitaniae* L17**	***C. albicans***	**Putative function of orthologs**	**log2FC (1)**	***Kannan et al.* (2)**	**Additional Information**
CLUG_01938_39 (MDR1)	*E0198_001392*	*C6_03170C_A (MDR1)*	Plasma membrane MDR/MFS multidrug efflux pump; methotrexate is preferred substrate; overexpression in drug-resistant clinical isolates confers fluconazole resistance; repressed in young biofilms; rat catheter biofilm induced	3.04	*upregulated*	
CLUG_03113 (CDR1)	*E0198_002139*	*C3_05220W_A (CDR1)*	Multidrug transporter of ABC superfamily; transports phospholipids in an in-to-out direction; induced by beta-estradiol, progesterone, corticosteroid, or cholesterol; Spider biofilm induced	1.41	*upregulated*	
CLUG_05825 (FLU1)	*E0198_005167*	*C7_01520W_A (FLU1)*	Multidrug efflux pump of the plasma membrane; MDR family member of the MFS (major facilitator superfamily) of transporters; involved in histatin 5 efflux; fungal-specific (no human/murine homolog)	0.78	*upregulated*	

CLUG_01594	*E0198_001720*	*CR_02020C_A (OPT1)*	Oligopeptide transporter; transports 3-to-5-residue peptides; alleles are distinct, one has intron; suppresses S. cerevisiae ptr2-2 mutant defects; induced by BSA or peptides; Stp3p, Hog1p regulated; flow model biofilm induced	0.72		
CLUG_04864	*E0198_004636*	*C1_09210C_A (SGE11)*	Putative transporter; slightly similar to the Sit1p siderophore transporter; Gcn4p-regulated; fungal-specific; induced by Mnl1p under weak acid stress	2.16		Not annotated to a *C. albicans* homolog in CGD. Homolog was identified using protein BLAST. Has 56% similarity to *S. cerevisiae* Azr1 which is a plasma membrane transporter of the major facilitator superfamily: involved in resistance to azole drugs such as ketoconazole and fluconazole
CLUG_01281 (MGD1)	*E0198_002004*	*C5_02860C_A (GRP2)*	NAD(H)-linked methylglyoxal oxidoreductase involved in regulation of methylglyoxal and pyruvate levels; regulation associated with azole resistance; induced in core stress response or by oxidative stress via Cap1, fluphenazine, benomyl (5)	3.96	*upregulated*	*CLUG_01281* is not mapped to the *C. albicans* homolog *GRE2* in CGD. Based on our phylogenetic analysis of *CLUG_01281* in *Biermann et al., 2019*, here we report *C5_02860C_A (GRE2)* as the *C. albicans* homolog of *CLUG_01281.*
CLUG_00542 (MRR1)	*E0198_000610*	*C3_05920W_A (MRR1)*	Putative Zn(II)2Cys6 transcription factor; regulator of MDR1 transcription; gain-of-function mutations cause upregulation of *MDR1* (a plasma membrane multidrug efflux pump) and multidrug resistance; Hap43-induced	1.2	*upregulated*	

CLUG_02139	*E0198_001225*	*CR_04960C_A (CRG1)*	Methyltransferase involved in sphingolipid homeostasis, methylates a drug cantharidin; decreased expression in hyphae compared to yeast; expression regulated during planktonic growth; flow model biofilm induced; Hap43-repressed gene	0.76		
CLUG_04865	*E0198_004635*	*#N/A*	No *C. albicans* ortholog. 57% identical to a putative major facilitator protein in *Candida auris* B8441 (B9J08_002249)	1.87		
CLUG_01282	*E0198_002003*	*C2_06870C_A (PST1)*	Flavodoxin-like protein involved in oxidative stress protection and virulence; putative 1,4-benzoquinone reductase; hyphal-induced; regulated by Cyr1, Ras1, Efg1, Nrg1, Rfg1, Tup1; Hap43-induced; Spider biofilm induced	2.93	*upregulated*	
CLUG_01393	*E0198_001905*	*C5_02860C_A (GRP2)*	Protein similar to dihydroflavonol-4-reductases; *GRP1*	5.79		Not annotated to a *C. albicans* homolog in CGD. Homolog was identified using protein BLAST.
CLUG_03198	*E0198_002873*	*C1_08330C_A (ADH2)*	Alcohol dehydrogenase; soluble in hyphae; expression regulated by white-opaque switching; regulated by Ssn6; indued by Mnl1 in weak acid stress; protein enriched in stationary phase yeast cultures; Spider biofilm induced	2.85	*upregulated*	
CLUG_02157	*E0198_001208*	*C1_08330C_A (ADH2)*	Alcohol dehydrogenase; soluble in hyphae; expression regulated by white-opaque switching; regulated by Ssn6; indued by Mnl1 in weak acid stress; protein enriched in stationary phase yeast cultures; Spider biofilm induced	8.49	*upregulated*	

CLUG_04593	*E0198_003888*	*C3_06860C_A*	Putative xylose and arabinose reductase; flow model biofilm induced; Spider biofilm repressed	0.72		Description changed from *Demers et al.* (1), induced by MMS Sc
CLUG_04429	*E0198_004040*	*#N/A*	No *C. albicans* ortholog. Similar to B9J08_003286 in C. auris (34% identical). No putative function.	0.79	*upregulated*	
CLUG_05005	*E0198_004530*	*C4_01840C_A*	Putative dienelactone hydrolase; protein abundance is affected by URA3 expression in the CAI-4 strain background; protein present in exponential and stationary growth phase yeast cultures; rat catheter biofilm repressed	3.26	*upregulated*	Not annotated to a *C. albicans* homolog in CGD. Homolog was identified using protein BLAST. Description changed from Demers *et al*. (1)
CLUG_00700	*E0198_000469*	*C1_00530C_A*	Protein similar but not orthologous to S. cerevisiae Bul1; a protein involved in selection of substrates for ubiquitination; mutants are viable; macrophage/pseudohyphal-induced; rat catheter biofilm induced	1.2		
CLUG_01574	*E0198_001732*	*#N/A*	Hypothetical protein with 48% identity to CJJ07_003908 in Candida auris strain B11243.	1.48		
CLUG_02042	*E0198_001302*	*C1_09150W_A (AOX2)*	Alternative oxidase; cyanide-resistant respiration; induced by antimycin A, oxidants; growth; Hap43, chlamydospore formation repressed; rat catheter, Spider biofilm induced; regulated in Spider biofilms by Bcr1, Tec1, Ndt80, Brg1	1.11		
CLUG_01897	*E0198_001437*	*C1_03190C_A (ECM33)*	GPI-anchored cell wall protein; mutants show cell-wall defects and reduced adhesion, host cell damage, and endocytosis; mutant infection is immunoprotective in murine model;	1.16		

			fluconazole-induced; caspofungin repressed			
CLUG_01020	*E0198_000159*	*C6_03790C_A (STL1/HGT10)*	Glycerol permease involved in glycerol uptake; member of the major facilitator superfamily; induced by osmotic stress, at low glucose in rich media, during cell wall regeneration; 12 membrane spans; Hap43p-induced gene	-0.76		
CLUG_01940	*E0198_001391*	*C1_08620W_A (CTR2)*	Putative low-affinity copper transporter of the vacuolar membrane; induced by nitric oxide; clade-associated gene expression; rat catheter and flow model biofilm induced	-0.85		
CLUG_02695	*E0198_002513*	*C5_01360W_A (CFL4)*	C-terminus similar to ferric reductases; induced in low iron; Sfu1-repressed; ciclopirox olamine induced; colony morphology-related gene regulation by Ssn6; Hap43-repressed; Sef1-regulated	-2.8		Not annotated to a *C. albicans* homolog in CGD. Homolog was identified using protein BLAST. Description changed from *Demers et al.*(1)
CLUG_02758	*E0198_002456*	*C2_08360C_A (OPT5)*	Oligopeptide transporter; fungal-specific (no human or murine homolog); induced by BSA, but not by tetrapeptide or pentapeptide	-0.64		
CLUG_04093	*E0198_003674*	*C7_00090C_A (CSA1)*	Surface antigen on elongating hyphae and buds; strain variation in repeat number; ciclopirox, filament induced, alkaline induced by Rim101; Efg1-, Cph1, Hap43-regulated; required for WT RPMI biofilm formation; Bcr1-induced in a/a biofilms	-2.02		

Supplemental Table 1B. GO slim analysis of *C. albicans* homologs of the genes in Table S1A was performed using Candida Genome Database (CGD). *C5_02860C_A* did not have a GO annotation.

GOID	GO term	Frequency	Gene(s)	Organism
6810	transport	9 out of 19 genes, 47.4%	*CFL4 FLU1 OPT5 CDR1 CTR2 C1_09210C_A MDR1 HGT10 OPT1*	*Candida albicans*
42221	response to chemical	8 out of 19 genes, 42.1%	*MRR1 AOX2 FLU1 CDR1 C1_09210C_A MDR1 C3_06860C_A PST1*	*Candida albicans*
6950	response to stress	5 out of 19 genes, 26.3%	*ECM33 CDR1 MDR1 C3_06860C_A PST1*	*Candida albicans*
19725	cellular homeostasis	5 out of 19 genes, 26.3%	*CRG1 CSA1 CDR1 CTR2 OPT1*	*Candida albicans*
44419	biological process involved in interspecies interaction between organisms	4 out of 19 genes, 21.1%	*CRG1 ECM33 MDR1 PST1*	*Candida albicans*
9410	response to xenobiotic stimulus	2 out of 19 genes, 10.5%	*CDR1 MDR1*	*Candida albicans*
50789	regulation of biological process	2 out of 19 genes, 10.5%	*MRR1 CFL4*	*Candida albicans*
5975	carbohydrate metabolic process	2 out of 19 genes, 10.5%	*ADH2 C3_06860C_A*	*Candida albicans*
42710	biofilm formation	2 out of 19 genes, 10.5%	*CSA1 ECM33*	*Candida albicans*
6091	generation of precursor metabolites and energy	2 out of 19 genes, 10.5%	*ADH2 AOX2*	*Candida albicans*
16070	RNA metabolic process	2 out of 19 genes, 10.5%	*MRR1 CFL4*	*Candida albicans*
8150	biological process	2 out of 19 genes, 10.5%	*C1_00530C_A C4_01840C_A*	*Candida albicans*
30447	filamentous growth	1 out of 19 genes, 5.3%	*ECM33*	*Candida albicans*
45333	cellular respiration	1 out of 19 genes, 5.3%	*AOX2*	*Candida albicans*
71555	cell wall organization	1 out of 19 genes, 5.3%	*ECM33*	*Candida albicans*

Supplemental Table 2. Strains

Strain	Lab #	Species	Parent	Relevant characteristics or genotype	Source
U04 (A04)	DH2949	*C. lusitaniae*		Clinical isolate, FLZ-resistant, *MRR1-Y813C*	(3, 4)
U04 *mrr1*Δ	DH3306	*C. lusitaniae*	U04	*mrr1*Δ::*NAT1*	(3)
U04 *mrr1*Δ + *MRR1-Y813C*	DH3613	*C. lusitaniae*	U04 *mrr1*Δ	*MRR1-Y813C-HygB*	(5)
U04 *mrr1*Δ + *MRR1-L1191H+Q1197** (*L1Q1**)	DH3626	*C. lusitaniae*	U04 *mrr1*Δ	*MRR1-L1191H+Q1197*-HygB*	(5)
U04 *mrr1*Δ + *MRR1-ancestral*	DH3611	*C. lusitaniae*	U04 *mrr1*Δ	*MRR1-ancestral-HygB*	(1)
U04 *mrr1*Δ + *MRR1-L1191H*	DH3630	*C. lusitaniae*	U04 *mrr1*Δ	*MRR1-L1191H-HygB*	(1)
U04 *mrr1*Δ + *HF-MRR1-Y813C*	DH4076	*C. lusitaniae*	U04 *mrr1*Δ	6xHis-3xFLAG-*MRR1-Y813C-HygB*	This study
U04 *mrr1*Δ + *HF-MRR1-L1191H+Q1197** (*L1Q1**)	DH3628	*C. lusitaniae*	U04 *mrr1*Δ	6xHis-3xFLAG-*MRR1-L1191H+Q1197*-HygB*	This study
U04 *mrr1*Δ + *HF-MRR1-ancestral*	DH3898	*C. lusitaniae*	U04 *mrr1*Δ	6xHis-3xFLAG-*MRR1-ancestral-HygB*	This study
U04 *mrr1*Δ + *MRR1-Y813C mdr1*Δ	DH3615	*C. lusitaniae*	U04 *mrr1*Δ + *MRR1-Y813C*	*MRR1-Y813C-HygB mdr1*Δ::*NAT1*	(1)
U04 *mrr1*Δ + *MRR1-Y813C* cdr1Δ	DH3678	*C. lusitaniae*	U04 *mrr1*Δ + *MRR1-Y813C*	*MRR1-Y813C-HygB cdr1*Δ::*NAT1*	This study

U04 *mrr1*Δ + *MRR1-Y813C* *flu1*Δ	DH3662	*C. lusitaniae*	U04 *mrr1*Δ + *MRR1-Y813C*	*MRR1-Y813C-HygB* *flu1*Δ::*NAT1*	This study
U04 *mdr1*Δ *cdr1*Δ	DH4778	*C. lusitaniae*	U04 *mdr1*Δ	*mdr1*Δ::*NAT1* *cdr1*Δ::*HygB*	This study

Plasmids in *E. coli* (DH5α)

Strain	Plasmid	Lab #	Relevant characteristics or genotype	Source
DH5α	pMQ30-*MRR1-L1191H+Q1197**	DH3829	pMQ30-$MRR1^{L1191H+Q1197*}$-*HygB* complementation, Gent^R	(5)
DH5α	pMQ30-*MRR1-ancestral*	DH3830	pMQ30-$MRR1^{ancestral}$- *HygB* complementation, Gent^R	(1)
DH5α	pMQ30-*MRR1-Y813C*	DH3831	pMQ30-$MRR1^{Y813C}$- *HygB* complementation, Gent^R	(5)
DH5α	pMQ30- 6His-3FLAG-*MRR1-L1191H+Q1197**	DH3877	pMQ30-*MRR16His3Flag*-$^{L1191H+Q1197*}$-*HygB* complementation, Gent^R	This study
DH5α	pMQ30-6His-3FLAG-*MRR1-ancestral*	DH3898	pMQ30-*MRR16His3Flag*-^{ancestral}- *HygB* complementation, Gent^R	This study
DH5α	pMQ30-6His-3FLAG-*MRR1-Y813C*	DH3963	pMQ30-*MRR16His3Flag*-^{Y813C}- *HygB* complementation, Gent^R	This study
DH5α	pNAT	DH2664	TEF1p-*NAT1*, Amp^R	(6)
DH5α	pYM70	DH3352	TEF2p-*HygB*, Amp^R	(7)
DH5α	pMQ30	DH2620	Plasmid that replicates in *S. cerevisiae* and *E. coli*, using uracil or gentamycin selection, respectively.	(8)
BL21(DE3)	pET51b-*MRR1*(1-196)	DH3603	pET51b-*MRR1*(1-196)-10xHis, Amp^R	This study

REFERENCES

1. Demers EG, Stajich JE, Ashare A, Occhipinti P, Hogan DA. 2021. Balancing Positive and Negative Selection: *In Vivo* Evolution of *Candida lusitaniae MRR1*. mBio 12.
2. Kannan A, Asner SA, Trachsel E, Kelly S, Parker J, Sanglard D. 2019. Comparative Genomics for the Elucidation of Multidrug Resistance in *Candida lusitaniae*. mBio 10:e02512-19.
3. Demers EG, Biermann AR, Masonjones S, Crocker AW, Ashare A, Stajich JE, Hogan DA. 2018. Evolution of drug resistance in an antifungal-naive chronic *Candida lusitaniae* infection. Proceedings of the National Academy of Sciences 115:12040-12045.
4. Grahl N, Demers EG, Crocker AW, Hogan DA. 2017. Use of RNA-Protein Complexes for Genome Editing in Non-albicans *Candida* Species. mSphere 2.
5. Biermann AR, Demers EG, Hogan DA. 2021. Mrr1 regulation of methylglyoxal catabolism and methylglyoxal-induced fluconazole resistance in *Candida lusitaniae*. Molecular Microbiology 115:116-130.
6. Min K, Ichikawa Y, Woolford CA, Mitchell AP. 2016. *Candida albicans* Gene Deletion with a Transient CRISPR-Cas9 System. mSphere 1:e00130-16.
7. Basso LR, Jr., Bartiss A, Mao Y, Gast CE, Coelho PS, Snyder M, Wong B. 2010. Transformation of *Candida albicans* with a synthetic hygromycin B resistance gene. Yeast 27:1039-48.
8. Shanks RM, Caiazza NC, Hinsa SM, Toutain CM, O'Toole GA. 2006. *Saccharomyces cerevisiae*-based molecular tool kit for manipulation of genes from gram-negative bacteria. Appl Environ Microbiol 72:5027-36.

Supplemental Table 3. Primers

Name	Description	Sequence
ED207	Forward to amplify 6xHIS-3xFLAG for *HF-MRR1* complement construct	GGAAACTTCATTACTAAAGATGATGCACCACCACCACCACCAC
ED208	Reverse to amplify 6xHIS-3xFLAG for *HF-MRR1* complement construct	TCGTTTTGGGTCTGAACCTCCTTGTCATCGTCATCTTTATAATC
ED103	Forward to amplify *MRR1* 5' flank w/ homology to pMQ30	TTTTCCCAGTCACGACGTTGTAAAACGACGGCCgcggccgcAAGGC GTGTCCTTCATGTT
ED206	Reverse to amplify *MRR1* gene with start ATG w/ homology to 6xHIS-3xFLAG	GTGGTGGTGGTGGTGGTGCATCATCTTTAGTAATGAAGTTTCC
ED209	Forward to amplify *MRR1* gene from 2nd codon w/ homology to 6xHIS-3xFLAG	GATTATAAAGATGACGATGACAAGGAGGTTCAGACCCAAAACGA
ED132	Reverse targeted to internal region of *MRR1* for *HF-MRR1* construct generation	CCAAATAAGACGCAAGCCGTAATCTCGCTTC
ED125	Forward to validate *HF-MRR1* in *C. lusitaniae*	GAAAAAGAAGCCAGCAGACC
ED126	Reverse to validate *HF-MRR1* in *C. lusitaniae*	GGGTAAAGCCATTGCAGAC
ED123	Reverse HygB internal, for validation	CATAACCTCTACCACCAACATC
ED124	Forward HygB internal, for validation	GCTCAAGGTAGATGTGATGC
ED191	Forward to amplify NAT for MMEJ-based *CDR1* knockout generation	GGTTGGCACATGGCCCATTTGTAGAAGGGTTTTTTCTTGTTTTAAG CCGACTATCCTCTTATTTATTCCGTTTTCCCAGTCACGACGTT
ED192	Reverse to amplify NAT for MMEJ-based *CDR1* knockout generation	AGTGAAGACAACTTTCATACTCTTTGTTCTTTTATTTCCTCAAGTGG CTGTACGTCAATAGCGAATACGCGTGGAATTGTGAGCGGATA
ED195	Forward for *CDR1* mutant validation	AACGCCTATGTTTTTAGCCG
ED196	Reverse for *CDR1* mutant validation	TAGTATGGATCACCAGTTCC
ED166	Forward to amplify NAT for MMEJ-based *FLU1* knockout generation	GATTTGTACAAAGAAGCATCTCATTCGACCGAGCCTTTGCCGCCA ATGGGGAGGAGACAAGGAGTATCCTgacatggaggcccagaatac
ED167	Reverse to amplify NAT for MMEJ-based *FLU1* knockout generation	TACTATCCTAGAATTTTCCTAACGAGTCAAAATTGAAATACTAGAAT GCTCATGAAATCGGATCATGAAGcagtatagcgaccagcattc
ED161	Forward for *FLU1* mutant validation	GGCATAGCAATTAGAGTAGC
ED162	Reverse for *FLU1* mutant validation	GACACCAAGAGAGTTGAGTC
pNATback	Forward NAT internal, for validation	CGA TGG TAC TGC TTC CGA TGG

NG_087	Reverse NAT internal, for validation	GAA GTT CCA GTT GAT CCA CCA TTG A
CDR1 crRNA #1	crRNA targeting 5' of *CDR1* (for *CDR1* MMEJ knockout)	TTATTCCGAGCGCTATACAA
CDR1 crRNA #2	crRNA targeting 3' of *CDR1* (for *CDR1* MMEJ knockout)	CTGGACATGAAATGTAGCTG
FLU1 crRNA #1	crRNA targeting 5' of *FLU1* (for *FLU1* MMEJ knockout)	CCCTGATCTGGGAGAAGCGG
FLU1 crRNA #2	crRNA targeting 3' of *FLU1* (for *FLU1* MMEJ knockout)	GTTGCGACAGGTATCTTTAA
NAT crRNA	crRNA for NAT (for HF-*MRR1* complementation)	GGG AAA ACC TTA GTC AAT GG

Supplemental Table 4. *MDR1* and *CDR1* gene IDs and start site coordinate information used for sequence retrieval for FIMO analysis

MDR1 co-ordinates

Accession	Species	Strains	Chromosome number	*MDR1* gene ID	*MDR1* start coordinate
GCA_003675555.2	*Clavispora lusitaniae*	L17	QOBI02000002.1	E0198_001392	758311
GCA_032599225.1	*Clavispora lusitaniae*	AR0398	JAUHPU010000001.1	Annotation not present, identified using pBLAST	1448371
GCA_032599145.1	*Clavispora lusitaniae*	79-1	JAUHPN010000003.1		136476
GCA_032599085.1	*Clavispora lusitaniae*	76-31	JAUHPM010000001.1		1451792
ASM383v1	*Clavispora lusitaniae*	ATCC 42720	CH408077.1	CLUG_01938_39	1458215
ASM18296v3	*Candida albicans*	SC5314	CP017628.1	CAALFM_C603170CA	666383
ASM18276v2	*Candida parapsilosis*	CDC317	HE605204.1	CPAR2_301760	409128
ASM1677213v1	*Candidozyma auris*	B11205	CP060339.1	CJI82_00130	296594

CDR1 co-ordinates

Accession	Species	Strains	Chromosome number	*CDR1* gene ID	*CDR1* start coordinate
GCA_003675555.2	*Clavispora lusitaniae*	L17	QOBI02000003.1	E0198_002139	198154
ASM383v1	*Clavispora lusitaniae*	ATCC 42720	CH408078.1	CLUG_03113	1683175
ASM18296v3	*Candida albicans*	SC5314	CP017625.1	C3_05220W_A	1146045
ASM18276v2	*Candida parapsilosis*	CDC317	HE605208.1	CPAR2_405290	1191166
GCA_002759435.3	*Candidozyma auris*	*B8441	CM076439.1	B9J08_02123 (old_locus_tagB9J08_00016)	348928

**CDR1* was not annotated in the Clade I *C. auris* B11205 strain, thus the Clade I *C. auris* B8441 was used for motif analysis of *CDR1* upstream region.

Supplemental Table 5. Genes used for FIMO analysis to evaluate cMBM enrichment upstream of Mrr1-regulated genes in S2C.

Group 1 – The 25 direct targets of Mrr1 (Fig. 4A & Supplemental Table 1A)

Group 2# – Genes that were differentially expressed when Mrr1 was constitutively active but had no CUT&RUN peak in their 1kb intergenic region (Supplemental File 2).

Group 3# – *C. lusitaniae* genes that neither had a CUT&RUN peak in their intergenic region nor were differentially expressed when Mrr1 was constitutively active.

Genes were randomly selected. Only genes annotated in both *C. lusitaniae* ATCC 42720 and L17 were used.

Group 1	Group 2	Group 3
CLUG_01938_39	*CLUG_04991*	*CLUG_03821*
CLUG_03113	*CLUG_02155*	*CLUG_03207*
CLUG_05825	*CLUG_04043*	*CLUG_04482*
CLUG_01594	*CLUG_01978*	*CLUG_02246*
CLUG_04864	*CLUG_04007*	*CLUG_05785*
CLUG_01281	*CLUG_00191*	*CLUG_01018*
CLUG_00542	*CLUG_00417*	*CLUG_04692*
CLUG_02139	*CLUG_05892*	*CLUG_02912*
CLUG_04865	*CLUG_02884*	*CLUG_03712*
CLUG_01282	*CLUG_05798*	*CLUG_05113*
CLUG_01393	*CLUG_04243*	*CLUG_02632*
CLUG_03198	*CLUG_02052*	*CLUG_04219*
CLUG_02157	*CLUG_03835*	*CLUG_02227*
CLUG_04593	*CLUG_02761*	*CLUG_00743*
CLUG_04429	*CLUG_02387*	*CLUG_01752*
CLUG_05005	*CLUG_00669*	CLUG_04822
CLUG_00700	*CLUG_01106*	*CLUG_04651*
CLUG_01574	*CLUG_02778*	*CLUG_05056*
CLUG_02042	*CLUG_00422*	*CLUG_00536*
CLUG_01897	*CLUG_01845*	*CLUG_00156*
CLUG_01020	*CLUG_04748*	*CLUG_01267*
CLUG_01940	*CLUG_02395*	*CLUG_04911*
CLUG_02695	*CLUG_01565*	*CLUG_02372*
CLUG_02758	*CLUG_00983*	*CLUG_04160*
CLUG_04093	*CLUG_04838*	*CLUG_00751*
